# Comparative Population Genomics Reveal the Determinants of Genome Erosion in Two Sympatric Neotropical Falcons

**DOI:** 10.1111/mec.17686

**Published:** 2025-02-03

**Authors:** Nicolas Dussex

**Affiliations:** ^1^ Department of Population Analysis and Monitoring Swedish Museum of Natural History Stockholm Sweden

**Keywords:** bottleneck, demography, genetic load, genome erosion, inbreeding

## Abstract

Studying genetic diversity in endangered species has become an important component of conservation science over the past decades. Thanks to recent developments in sequencing technologies and bioinformatics, genetic parameters of conservation relevance such as neutral and functional genome‐wide variation are now routinely estimated. Since inbreeding and deleterious mutations represent significant threats to small and declining populations, assessing the dynamics of these parameters has received particular attention in many recent conservation genomics studies. In this issue of Molecular Ecology, Martin et al. analyse the genomes of two Neotropical falcon species to assess the impact of their contrasting population histories on genome‐wide diversity. They show that the Orange‐breasted falcon which has had a low long‐term population size and has experienced recent population bottlenecks is more inbred but has relatively fewer deleterious variations compared to its sister taxon, the Bat falcon, which is characterised by a larger long‐term population size. This study not only provides insights into the role of past demography on the dynamics of deleterious variation in two species with contrasting population histories but also highlights the increasing importance of comparative approaches in population and conservation genomics.

Martin et al. (2024) examined the effects of demographic history on the genome‐wide variation of two Neotropical falcon sister species with overlapping ranges. The authors sequenced 21 falcon genomes to reconstruct past demography and to estimate genome‐wide diversity, inbreeding and the amount of deleterious variation (i.e., genetic load), which are indices commonly used to assess the threat of genome erosion (Bertorelle et al. 2022). Small populations are particularly prone to genome erosion through high inbreeding and accumulation of genetic load, which can reduce fitness and increase the risk of population extinction. However, long‐term small population size and inbreeding can facilitate the reduction of a portion of the genetic load through natural selection, a process referred to as purging. In contrast, large populations will retain a greater proportion of genetic load hidden as heterozygous recessive mutations not affecting individual fitness (Hedrick and Garcia‐Dorado 2016). The comparison of patterns of genome erosion in species with contrasting demographic histories, as done by Martin et al. (2024) can thus reveal important determinants of inbreeding and deleterious variation.

The authors first examined the population structure of the two species, which revealed a shared population clustering between Central and South America, with a clear split North and South of Panama. Next, they reconstructed their population histories using a coalescent approach and estimation of effective population size (N_e_) over the past 10 million to 10,000 years. Their reconstructions revealed that the Orange‐breasted falcon had a low long‐term N_e_ compared to the majority of Bat falcon individuals, whereas those from Central America shared a similar N_e_ to Orange‐breasted falcons. Overall estimates of inbreeding (F_ROH_) were ~10 times higher in Orange‐breasted falcons compared to Bat falcons (Figure 1). However, the authors also note that both species show evidence of recent declines dating back to ~180 to 300 years ago, likely resulting from habitat reduction and human persecution. When estimating genetic load, Martin et al. (2024) found a ~threefold reduction in the proportion of highly deleterious variants in heterozygous state (i.e., masked load or inbreeding load), but a ~1.3‐fold higher proportion of load expressed as homozygous (i.e., realised load or drift load; Bertorelle et al. 2022; Dussex et al. 2023) in Orange‐breasted falcons, consistent with their higher inbreeding (Figure 1). Additionally, they found an excess in the frequency of non‐synonymous alleles in orange‐breasted falcons. While not strongly damaging, these alleles can potentially impact protein function and effectiveness and thus be considered as mildly deleterious. Finally, consistent with a smaller population size, there was a higher proportion of deleterious variants shared among orange‐breasted falcons compared to Bat falcons. Taken together, these results suggest that long‐term small population size may have facilitated the reduction of a portion of genetic load via a purging in orange‐breasted falcons, whereas a more recent increase in inbreeding contributed to a higher expression of this load.

**FIGURE 1:**
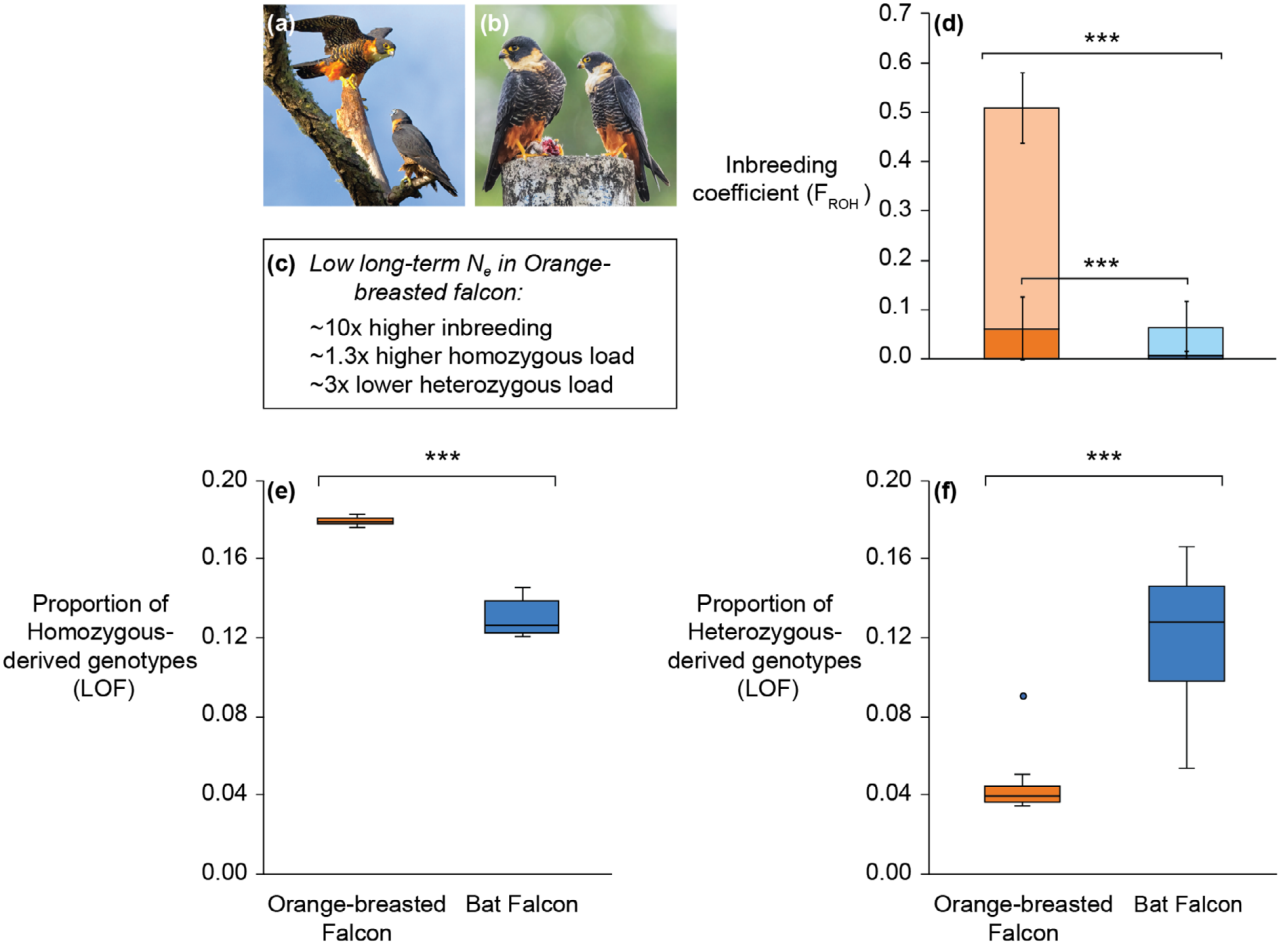
Inbreeding and genetic load in Orange‐breasted and Bat falcons. (a) Orange‐breasted (Falco deiroleucus) and, (b) Bat (Falco rufigularis) falcons. (c) Consequences of low long‐term Ne in Orange‐breasted falcon. (d) Inbreeding coefficient with the proportion of genome in runs of homozygosity (ROH) ≥ 100 kb (open bars) and ≥ 1 Mb (solid bars) among species (***p < 0.001). (e) proportion of homozygous‐derived and, (f) heterozygous‐derived Loss‐of‐Function (LOF) alleles. Horizontal lines within boxplots and bounds of boxes/whiskers represent medians and standard quartile ranges, respectively (***p < 0.001). Photo credits: (a) Robert Berry, (b) Bill Wood, Cornell Lab of Ornithology.

The pattern observed in orange‐breasted falcons is not uncommon in wild and inbred threatened species with several examples of reduction in genetic load. This pattern also highlights the dynamic nature of genetic load, which is influenced by both genetic drift and natural (i.e., purifying) selection (Dussex et al. 2023). Indeed, long‐term low population size and high inbreeding can facilitate the purging of highly deleterious variation by selection, whereas mildly or slightly deleterious variation may increase in frequency or drift to fixation more easily due to their lower selection coefficients (Hedrick and Garcia‐Dorado 2016; Figure 2). While deleterious mutations are mostly recessive, when in high frequency, they can reduce population survival through additive effects if they are associated with genes underpinning complex polygenic traits (Bertorelle et al. 2022; Dussex et al. 2023). For instance, several inbred species have purged a portion of their highly deleterious load while still showing a potential risk of inbreeding depression, via an excess in the frequency of mutations of mild to weak effects (e.g., Alpine Ibex; Grossen et al. 2020; Indian tigers; Khan et al. 2021). Consequently, in the long term, this type of load could increase the risk of extinction in orange‐breasted falcons, even though no negative effect on fitness has been observed yet.

**FIGURE 2:**
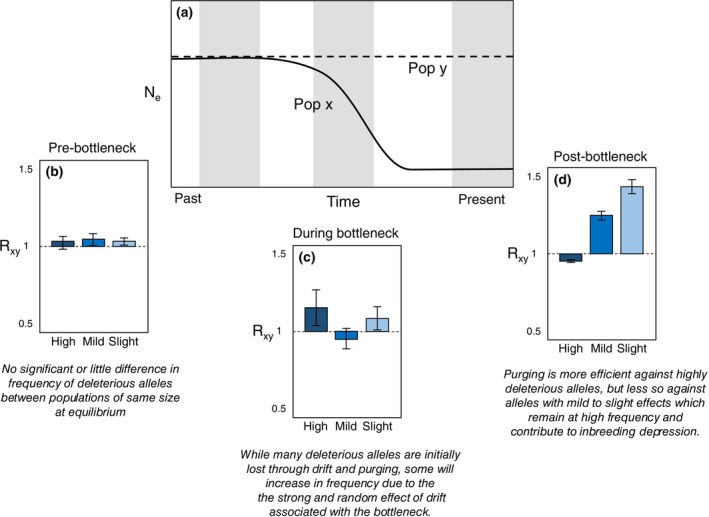
Conceptual depiction of the dynamics of genetic load at different stages of a demographic decline. (a) Past demographic trajectory showing effective population size (N_e_) through time in a declining (*x*) and stable (*y*) population with a large long‐term N_e_. (b–d) R_xy_ statistics for highly, mildly and slightly deleterious alleles at the pre‐bottleneck, during bottleneck and post‐bottleneck stages. The R_xy_ ratio compares the declining population *x* to the stable population *y*. R_xy_ < 1, =1 or > 1 corresponds to a deficit, no difference or an excess in allele frequency, respectively, in population *x* relative to population *y*. Whiskers depict ±1 SD.

Conservation biologists often consider genetic supplementation via enhanced natural gene flow or translocations as a means of species management, as it may induce a genetic rescue effect (Bell et al. 2019). Since orange‐breasted falcons show high inbreeding and possibly reduced adaptive potential, increasing gene flow among subpopulations may be beneficial. Yet, even though gene flow seems to overwhelmingly contribute to reducing inbreeding depression and increasing population survival in numerous taxa (Bell et al. 2019), new deleterious alleles may also be introduced via gene flow. Martin et al. (2024) suggestion that the much higher number of deleterious variants in one orange‐breasted falcon from Panama may be the result of admixture between birds from Panama and South America is consistent with this hypothesis. They also argue that while orange‐breasted falcons do not show evidence of reduction in fitness yet, the risk of inbreeding depression resulting from future admixture is worth considering. For instance, the famous case of the Isle Royale wolf indicates that a rapid population collapse can occur with only one breeding migrant introducing new deleterious variation into an inbred population (Robinson et al. 2019).

A growing number of conservation genetics studies have examined the dynamics of genetic load in small populations and have highlighted the value of genomics as an essential component of the conservation biology toolbox. However, several knowledge gaps remain. First, while correlations between the proportion of deleterious variations and certain fitness traits can improve our understanding of the genetic basis of inbreeding depression (e.g., Hasselgren et al. 2024), measuring fitness effects is often challenging and typically requires an experimental setting. Secondly, few studies have used gene expression data (e.g., RNA) to test whether there is a correlation between gene expression and the amount of deleterious mutations. Testing whether selection is stronger in highly expressed genes would thus improve our understanding of the interplay between genetic expression and selection (i.e., purging). Third, while forward‐in‐time simulations are increasingly used in conservation genetics studies to validate the interpretation of empirical data (Kyriazis, Robinson, and Lohmueller 2023), only a few studies have fully exploited their predictive power. These could, for instance, inform conservation actions by determining the number of immigrants or frequency of immigration events required to ensure a certain level of maintenance of genetic diversity, or conversely, to reduce the proportion of deleterious variation introduced in a recipient population. This would, in turn, facilitate the creation of corridors for dispersal or even allow the selection of the most suitable individuals for translocations.

The study of Martin et al. (2024) provides the ideal framework to assess the effect of demographic fluctuations on genetic variation and demonstrates that past demography is indeed a strong determinant of genetic load in wild populations. Importantly, their study highlights the increasing value of comparative approaches in the current biodiversity crisis and will without a doubt stimulate a number of similar studies in other endangered species. Applying a comparative framework to broader taxonomic, geographical and ecological levels will certainly improve our understanding of the genomic effects of population bottlenecks but will also allow us to evaluate the roles of different ecologies, life‐history traits and behaviours on the intensity of and resilience to genome erosion.

## Author Contributions

The author takes full responsibility for this article.

## Conflicts of Interest

The author declares no conflicts of interest.

## Data Availability

The author has nothing to report.
